# Beta-blocker administration within 24 hours after admission to the intensive care unit and mortality in critical heart failure patients: a retrospective analysis from the MIMIC-IV database

**DOI:** 10.3389/fphar.2025.1514138

**Published:** 2025-02-26

**Authors:** Linfeng Xie, Jing Chen, Yuanzhu Li, Gang Liu, Jian Shen, Xiang Li, Yuan Yang, Yintao Chen, Suxin Luo, Bi Huang

**Affiliations:** Department of Cardiology, The First Affiliated Hospital of Chongqing Medical University, Chongqing, China

**Keywords:** beta blockers, critical, heart failure, mortality, MIMIC-IV

## Abstract

**Background:**

It remains poorly understood whether early use of beta-blockers could provide a survival advantage in patients with critical heart failure (HF) .

**Methods:**

This retrospective study was conducted using the American Medical Information Mart for Intensive Care (MIMIC)-IV database. Study participants were critical HF patients who were divided into two groups: within 24-hour use of beta-blockers group and no use of beta-blockers group. The primary study endpoints were 7-day, 30-day, and 360-day all-cause mortality.

**Results:**

Out of the 10,184 patients diagnosed with critical HF, after propensity score match (PSM), 7352 patients were recruited and were divided into within 24-h use of beta-blockers group (n = 3676) and no beta blockers group (n = 3676). The 7-day, 30-day, and 360-day all-cause mortality were significantly higher in the no beta blockers group (7-day: 10.3% vs 5.5%; 30-day: 21.4% vs 15.7%; 360-day: 40.0% vs 35.3%; all p < 0.001). Kaplan–Meier analyses showed that the cumulative incidence of 7-day, 30-day, and 360-day all-cause mortality were significantly higher in the no beta blockers group (all log-rank p < 0.001). After PSM, Cox proportional hazards analyses revealed that beta blockers administration within 24 h of admission to intensive care unit (ICU) was independently associated with decreased 7-day (HR = 0.52 95%CI: 0.44, 0.62, p < 0.001), 30-day (HR = 0.70 95%CI: 0.63, 0.78, p < 0.001), and 360-day (HR = 0.83 95%CI: 0.77, 0.89, p < 0.001) all-cause mortality.

**Conclusion:**

Administration of beta blockers within 24 h after admission to ICU was associated with reduced risk of mortality in critical HF patients. However, prospective randomized controlled trials are needed to confirm our findings due to the retrospective nature of the present study and the limitations of the MIMIC-IV database itself.

## 1 Introduction

Heart failure (HF) is the manifestation when a cardiovascular disease progresses into severe stage (McDonagh et al., 2023). In recent decades, advancements achieved in pharmacological treatment have significantly improved the prognosis of patients with chronic HF (McDonagh et al., 2023). Among the medications used to improve outcomes in chronic HF, especially in patients with HF and reduced ejection fraction (HFrEF), beta-blockers improved the prognosis by blocking sympathetic activity, reducing catecholamine release and heart rate, as well as improving cardiac remodeling (Kubon et al., 2011; Udelson, 2004). Currently, beta-blockers are the first-line treatment for chronic HF with reduced ejection fraction recommended by guidelines (McDonagh et al., 2023; Heidenreich et al., 2022).

Although the role of beta blockers in improving outcomes for patients with HFrEF is well-established (Fowler, 1997; Packer et al., 1996; Authors Anonymous, 1999a; Authors Anonymous, 1999b), consensus remains elusive on whether to continue the use of beta blockers in acute HF patients who have been previously treated or to initiate the beta blockers in those patients who have not used them before. Tamaki et al. reported that initiation of beta blockers at admission reduced in-hospital mortality in acute decompensated HF patients, regardless of left ventricular ejection fraction (LVEF) (Tamaki et al., 2021). A recent meta-analysis involving acute HF or cardiogenic shock patients demonstrated that early beta-blocker initiation provided a survival advantage, including the in-hospital composite endpoints, in-hospital all-cause mortality, discharge mortality, and rehospitalization (Sinardja et al., 2024). These studies highlight the clinical benefits of early use of beta blockers compared to their delayed administration in patients with acute HF.

However, critical HF patients present with severely impaired cardiac function, markedly reduced contractility, and significant systemic hypoperfusion (Crespo-Leiro et al., 2018). The negative inotropic effects of beta-blockers may further suppress cardiac contractility, potentially exacerbating cardiac dysfunction (Kubon et al., 2011; Tamaki et al., 2021). Additionally, beta blockers induce vasodilation, which can lead to a further decline in blood pressure (Niu and Qi, 2016). Consequently, these effects may disrupt the cardiovascular system’s compensatory mechanisms, thereby further impairing cardiac function and systemic perfusion. Nevertheless, early use of beta-blockers may provide potential benefits for such critically ill HF patients by inhibiting excessive sympathetic activation (Kubon et al., 2011; Tamaki et al., 2021). However, to date, no studies have evaluated whether critically ill HF patients could benefit from early administration of beta blockers. Therefore, the objective of this study was to evaluate whether beta blockers administration within 24 h after admission in an intensive care unit (ICU) would provide a survival advantage in critical HF patients.

## 2 Methods

### 2.1 Data source and study design

This retrospective study was performed to evaluate whether beta blockers (metoprolol, bisoprolol, and carvedilol) administration within 24 h of ICU admission would provide a survival advantage in critical HF patients. The data of this study were taken from the American Medical Information Mart for Intensive Care (MIMIC)-IV (version 3.0) database (Johnson et al., 2023), which is a publicly accessible clinical database containing 94,458 ICU stays and clinical outcomes after discharge between 2008 and 2022 at the Beth Israel Deaconess Medical Centre, Boston. One author, L.F.X, completed the Collaborative Institutional Training Initiative examination (Certification number: 57983166) and obtained permission to access this database. Individual patient consent was not needed because de-identification was performed in the MIMIC-IV database, and the study protocol was approved by the Ethics Committee of the First Affiliated Hospital of Chongqing Medical University, China and complied with the Declaration of Helsinki.

### 2.2 Study population

**FIGURE 1:**
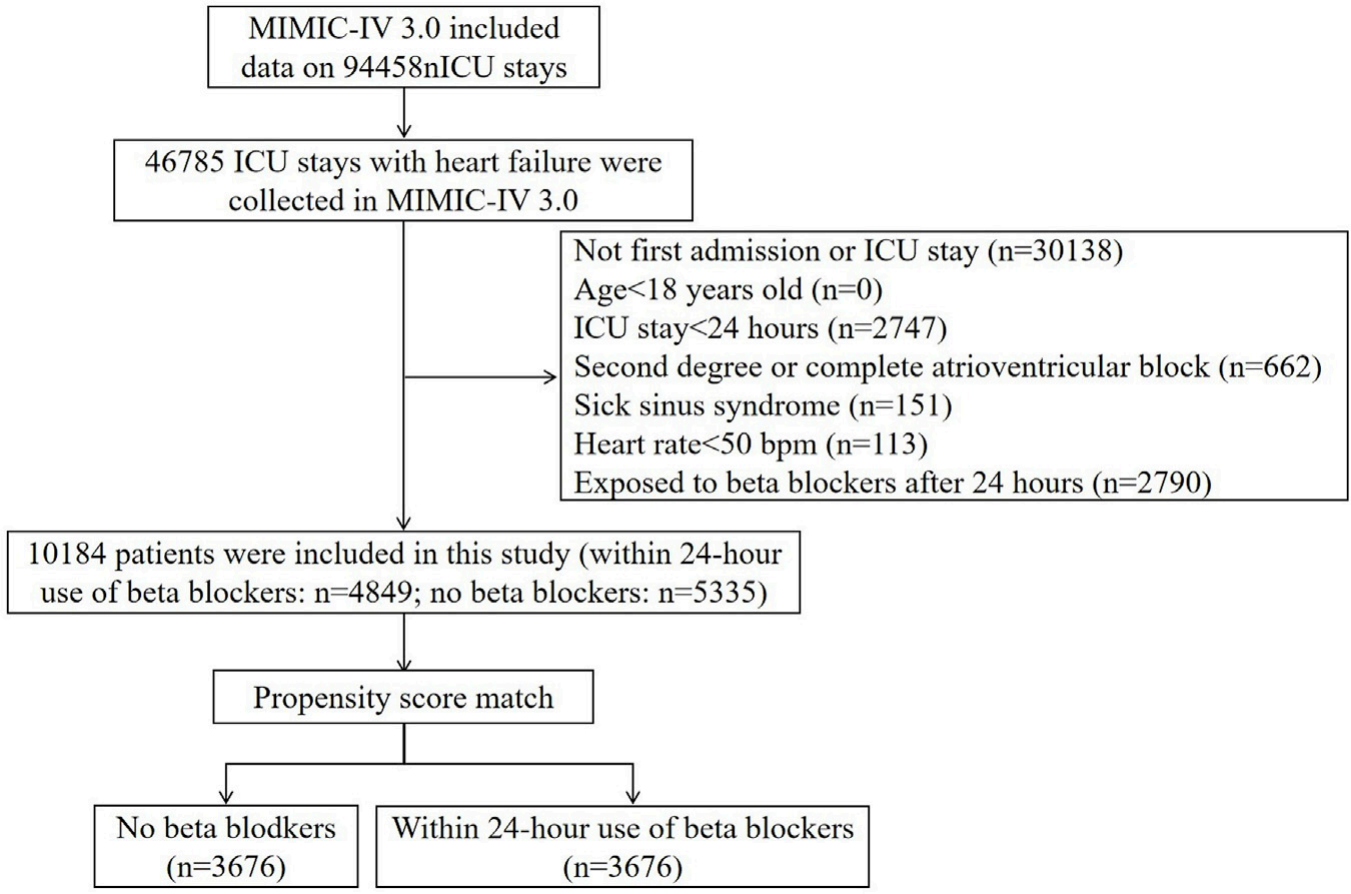
Flowchart of study participants. MIMIC: Medical Information Mart for Intensive Care.

The study population was critical HF patients as defined by the criteria of ICD-9 and ICD-10 codes. The exclusion criteria were as follows: 1) age <18 years old; 2) stayed less than 24 h in ICU; 3) patients with second-degree or complete atrioventricular block; 4) patients with sick sinus syndrome; 5) heart rate <50 bpm; 6) exposure to beta blockers after 24 h of ICU admission. For patients with multiple admissions to the ICU for HF, data from the first admission were extracted (Figure 1).

### 2.3 Data extraction

Using PostgresSQL software (version 13.7.2) and Navicat Premium software (version 16) through the execution of a Structured Query Language (SQL), author L.F.X extracted the data for this study, including demographic data, clinical data, clinical outcomes, and the results of laboratory test; The first results of laboratory test were extracted after ICU admission. Moreover, LVEF data were also extracted.

### 2.4 Study endpoints

The endpoints of this study were 7-day, 30-day, and 360-day all-cause mortality.

### 2.5 Statistical analysis

Continuous variables that follow a normal distribution are expressed as the mean and standard deviation (SD), and the differences between groups were tested with an independent sample t-test. The median and interquartile range (25%–75%) were reported for variables that do not follow a normal distribution and the Mann–Whitney U test was performed for the comparison between the two study groups. Categorical variables are expressed as numbers (percentage), and comparisons between groups were conducted by the chi-square test or Fisher’s exact test as appropriate.

Propensity score match (PSM) was conducted to balance the baseline characteristics between within 24-h use of beta blockers group and no beta blockers group. Thus, we used a logistic regression model to determine the PSM score for each patient and performed 1:1 matching for the two groups. After PSM, standardized mean difference (SMD) was performed to evaluate the remaining characteristics between the two groups.

The study endpoints were compared between the two groups. The cumulative incidence of 7-day, 30-day, and 360-day all-cause mortality was assessed by Kaplan–Meier analyses and the comparisons between the two groups were conducted by log-rank test. Cox proportional hazards analyses were performed to assess the association between beta blockers’ administration within 24 h of ICU admission and all-cause mortality.

The robustness of the effect of beta blockers administration within 24 h of ICU admission was evaluated using sensitivity analysis through comparing the data both before and after PSM and subgroup analysis. For analysis of the data before PSM, three models were constructed. Model 1 was unadjusted, model 2 adjusted for age and gender, and model 3 adjusted for age, gender, race, weight, systolic blood pressure, heart rate, respiratory rate, hematocrit, hemoglobin, platelet, white blood cell, red blood cell distribution, blood urea nitrogen, creatinine, potassium, cerebrovascular disease, chronic pulmonary disease, liver disease, diabetes, chronic kidney disease, cancer, acute myocardial infarction, atrial fibrillation, sepsis, and sofa score. Subgroup analysis was conducted to explore whether the impact of beta blocker administration within 24 h of ICU admission on all-cause mortality was consistent across different subgroups classified by age, gender, race, heart rate, systolic blood pressure, acute myocardial infarction, atrial fibrillation, chronic pulmonary disease, peripheral vascular disease, chronic kidney disease, LVEF, and different dosages and durations of different type of beta blockers. In this study, a 2-tailed p-value of <0.05 was considered statistically significant and all statistical analyses were carried out using SPSS statistical software, version 25.0 (IBM, United States), GraphPad Prism 8.4.3, and R version 4.1.2 (R Foundation).

## 3 Results

### 3.1 Patient characteristics

A total of 10184 eligible patients were included in this study (Figure 1). A total of 4849 patients received beta blockers within the first 24 h after ICU admission, among whom 4398 (90.7%) patients received metoprolol, 447 (9.2%) patients received carvedilol, and 4 (0.1%) patients received bisoprolol. The mean (SD) age of this cohort was 73.12 (13.46) years, and 5643 (55.41%) were male individuals.

**FIGURE 2:**
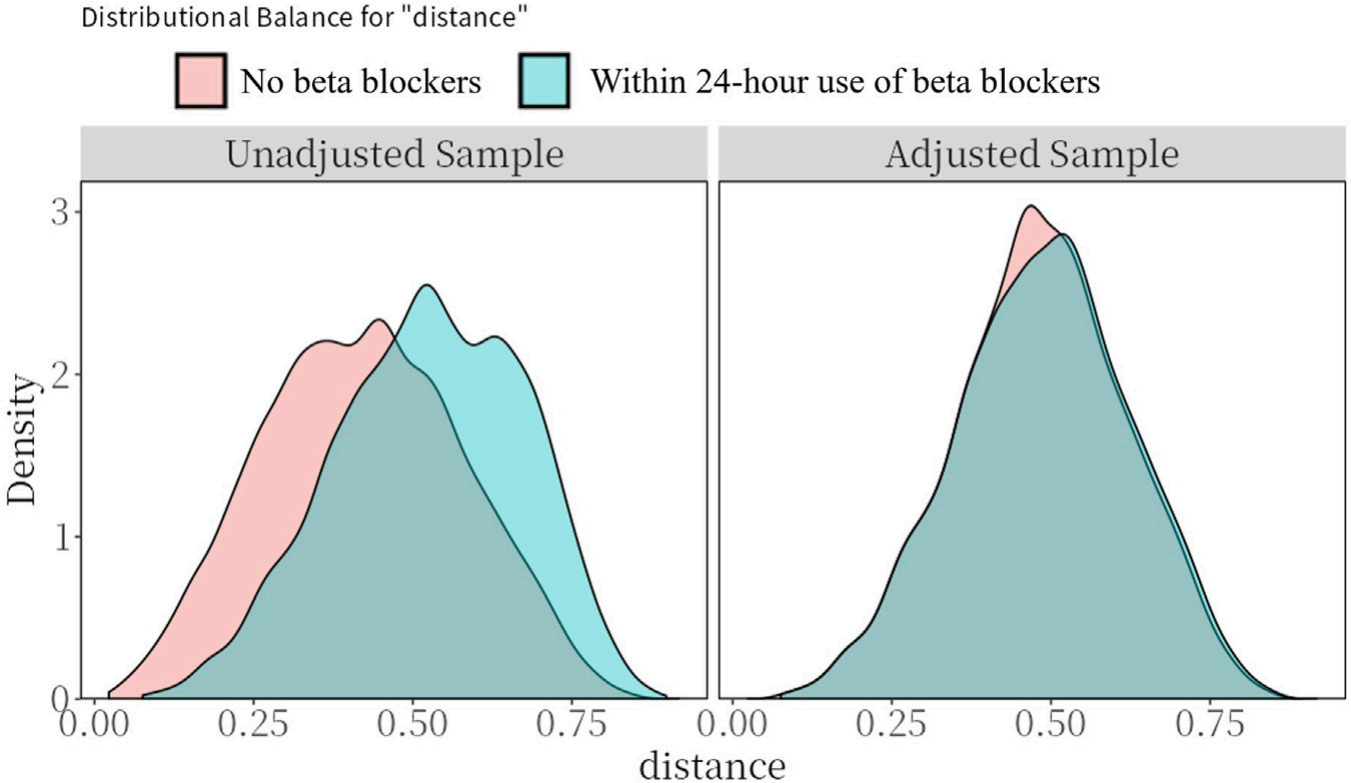
Preference score distributions. Greater overlap indicates that patients in the target and comparator populations are more similar in their likelihood of receiving the target treatment.

Table 1 shows the baseline characteristics between within 24 h use of beta blockers group and no beta blockers group. Before PSM, patients in within 24 h use of beta blockers group tended to be older and had a higher proportion of male, white individuals (all p < 0.05). As for the vital signs, patients who received early administration of beta blockers showed higher systolic blood pressure, heart rate, and SPO_2_, but a lower respiratory rate (all p < 0.05). In terms of comorbidities, patients in within 24 h use of beta blockers group had a higher proportion of acute myocardial infarction, atrial fibrillation, diabetes, primary hypertension, and cerebrovascular disease, but a lower proportion of chronic pulmonary disease, liver disease, chronic kidney disease, cancer, and sepsis (all p < 0.05). In addition, patients who received early administration of beta blockers had higher levels of hematocrit, hemoglobin, platelet, calcium, chlorine, and sodium, but had relatively lower red blood cell distribution width, potassium, blood urea nitrogen, and creatinine (all p < 0.05). Moreover, patients in within 24 h use of the beta blockers group had lower Sequential Organ Failure Assessment (SOFA) score (p < 0.001). After PSM, the baseline variables between the two groups were found comparable (all SMD <0.1, Table 1; Figure 2).

**TABLE 1:** Baseline characteristics of patients with critical heart failure patients before and after propensity score match.

	Before propensity score matching					After propensity score matching			
	Total (n = 10,184)	No beta blockers (n = 5335)	Within 24 h use of beta blockers (n = 4849)	p value	SMD	Total (n = 7352)	No beta blockers (n = 3676)	Within 24 h use of beta blockers (n = 3676)	SMD
Demographic characteristic									
Age, mean ± SD	73.12 ± 13.46	72.46 ± 14.09	73.83 ± 12.69	<0.001	0.108	73.73 ± 13.27	73.78 ± 13.63	73.67 ± 12.89	0.008
Male, n (%)	5,643 (55.41)	2863 (53.66)	2780 (57.33)	<0.001	0.074	4061 (55.24)	2029 (55.20)	2032 (55.28)	0.002
Race, white, n (%)	6,943 (68.18)	3574 (66.99)	3369 (69.48)	0.007	0.054	5027 (68.38)	2514 (68.39)	2513 (68.36)	0.001
Vital signs									
Systolic blood pressure, mean ± SD	122.76 ± 24.96	120.74 ± 25.53	124.99 ± 24.12	<0.001	0.176	123.94 ± 24.86	123.87 ± 25.42	124.01 ± 24.28	0.006
Heart rate, mean ± SD	89.44 ± 20.15	88.35 ± 19.53	90.64 ± 20.75	<0.001	0.111	89.33 ± 19.96	89.04 ± 19.75	89.62 ± 20.16	0.028
Respiratory rate, mean ± SD	20.26 ± 6.20	20.66 ± 6.39	19.81 ± 5.95	<0.001	0.142	20.27 ± 6.06	20.29 ± 6.07	20.25 ± 6.06	0.007
Spo2, mean ± SD	96.41 ± 4.25	96.23 ± 4.43	96.62 ± 4.03	<0.001	0.098	96.43 ± 4.06	96.45 ± 3.84	96.41 ± 4.27	0.009
Comorbidities									
Acute myocardial infarction, n (%)	1,559 (15.31)	738 (13.83)	821 (16.93)	<0.001	0.083	1122 (15.26)	564 (15.34)	558 (15.18)	0.005
Atrial fibrillation, n (%)	4,959 (48.69)	2,194 (41.12)	2,765 (57.02)	<0.001	0.321	3,713 (50.5)	1,843 (50.14)	1,870 (50.87)	0.015
Diabetes, n (%)	4,197 (41.21)	2,139 (40.09)	2,058 (42.44)	0.016	0.048	3,068 (41.73)	1,542 (41.95)	1,526 (41.51)	0.009
Primary hypertension, n (%)	2,584 (25.37)	1181 (22.14)	1403 (28.93)	<0.001	0.150	1885 (25.64)	938 (25.52)	947 (25.76)	0.006
Cerebrovascular disease, n (%)	1,526 (14.98)	699 (13.10)	827 (17.06)	<0.001	0.105	1104 (15.02)	556 (15.13)	548 (14.91)	0.006
Chronic pulmonary disease, n (%)	3,787 (37.19)	2,037 (38.18)	1750 (36.09)	0.029	0.044	2728 (37.11)	1,366 (37.16)	1,362 (37.05)	0.002
Liver disease, n (%)	983 (9.65)	683 (12.80)	300 (6.19)	<0.001	0.275	556 (7.56)	278 (7.56)	278 (7.56)	0.000
Chronic kidney disease, n (%)	3,815 (37.46)	2,063 (38.67)	1752 (36.13)	0.008	0.053	2,821 (38.37)	1,425 (38.76)	1,396 (37.98)	0.016
Cancer, n (%)	1,151 (11.3)	658 (12.33)	493 (10.17)	<0.001	0.072	810 (11.02)	393 (10.69)	417 (11.34)	0.021
Sepsis, n (%)	5,473 (53.74)	3,085 (57.83)	2,388 (49.25)	<0.001	0.172	3,850 (52.37)	1,933 (52.58)	1,917 (52.15)	0.009
Laboratory test									
Hematocrit, mean ± SD	33.23 ± 7.29	32.92 ± 7.58	33.56 ± 6.95	<0.001	0.092	33.39 ± 7.16	33.34 ± 7.38	33.44 ± 6.95	0.013
Hemoglobin, mean ± SD	10.74 ± 2.42	10.59 ± 2.49	10.91 ± 2.34	<0.001	0.136	10.80 ± 2.39	10.78 ± 2.45	10.82 ± 2.34	0.018
Platelet, M (Q₁, Q₃)	201.0 (147.0, 267.0)	200.0 (143.0, 267.0)	202.0 (151.0, 267.00)	0.009	0.047	204.0 (150.0, 271.0)	205.0 (151.0, 273.0)	202.0 (149.0, 269.0)	0.016
White blood cell, M (Q₁, Q₃)	10.50 (7.70, 14.70)	10.60 (7.50, 15.10)	10.50 (7.80, 14.20)	0.466	0.062	10.50 (7.70, 14.50)	10.40 (7.60, 14.70)	10.50 (7.80, 14.30)	0.014
RDW, mean ± SD	15.54 ± 2.43	15.84 ± 2.54	15.21 ± 2.26	<0.001	0.279	15.43 ± 2.30	15.45 ± 2.23	15.41 ± 2.37	0.018
Calcium, mean ± SD	8.54 ± 0.83	8.50 ± 0.88	8.59 ± 0.76	<0.001	0.116	8.57 ± 0.83	8.57 ± 0.88	8.56 ± 0.77	0.014
Chlorine, mean ± SD	101.59 ± 6.97	101.16 ± 7.37	102.05 ± 6.48	<0.001	0.138	101.64 ± 6.85	101.64 ± 7.11	101.64 ± 6.58	0.000
Sodium, mean ± SD	137.77 ± 5.51	137.59 ± 5.86	137.96 ± 5.09	<0.001	0.075	137.89 ± 5.44	137.91 ± 5.62	137.87 ± 5.24	0.007
Potassium, mean ± SD	4.43 ± 0.88	4.47 ± 0.94	4.38 ± 0.81	<0.001	0.111	4.41 ± 0.87	4.41 ± 0.89	4.41 ± 0.85	0.011
BUN, M (Q₁, Q₃)	27.00 (17.00, 43.00)	29.00 (18.00, 47.00)	25.00 (17.00, 39.00)	<0.001	0.259	26.00 (17.00, 43.00)	27.00 (18.00, 43.00)	26.00 (17.00, 42.00)	0.018
Creatinine, M (Q₁, Q₃)	1.20 (0.90, 1.90)	1.30 (0.90, 2.10)	1.20 (0.90, 1.70)	<0.001	0.232	1.20 (0.90, 1.90)	1.20 (0.90, 1.90)	1.20 (0.90, 1.80)	0.007
Glucose, M (Q₁, Q₃)	132.0 (108.0, 174.0)	131.0 (106.0, 174.0)	132.0 (109.0, 173.0)	0.168	0.007	133.0 (108.0, 174.0)	134.0 (108.0, 175.0)	133.0 (108.0, 173.3)	0.003
Sofa score, M (Q₁, Q₃)	1.00 (0.00, 3.00)	1.00 (0.00, 4.00)	1.00 (0.00, 3.00)	<0.001	0.249	1.00 (0.00, 3.00)	1.00 (0.00, 3.00)	1.00 (0.00, 3.00)	0.013

RDW, red blood cell distribution width; BUN, blood urea nitrogen.

### 3.2 Outcomes

**FIGURE 3:**
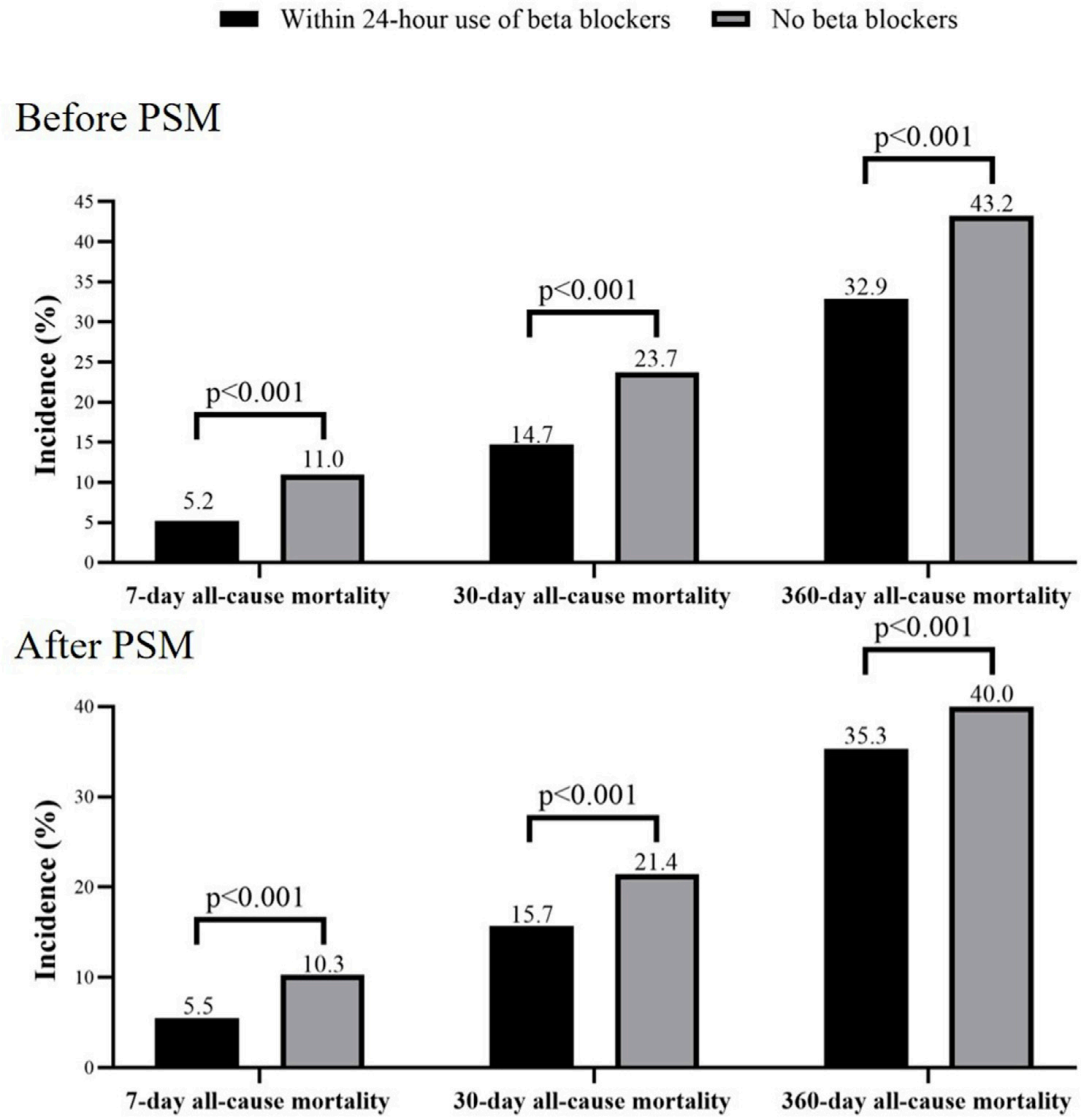
7-day, 30-day, and 360-day all-cause mortality in within 24-hour use of beta blockers group and no beta blockers group. PSM, propensity score match.

After PSM, the 7-day, 30-day, and 360-day all-cause mortality were significantly higher in no beta blockers group compared with within 24-h use of beta blockers group (7-day: 10.3% vs 5.5%; 30-day: 21.4% vs 15.7%; 360-day: 40.0% vs 35.3%; all p < 0.001, Figure 3).

**FIGURE 4:**
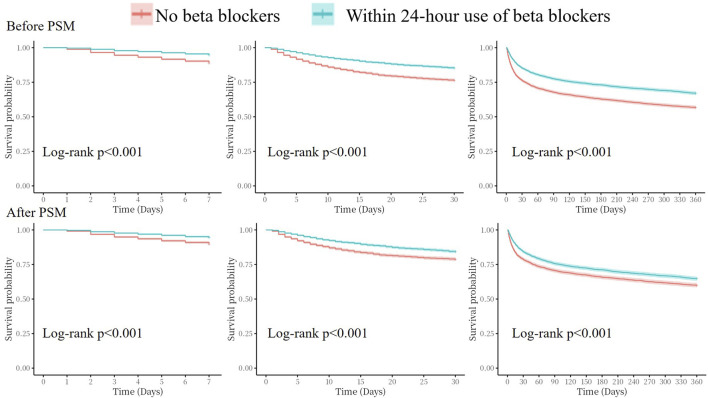
Kaplan–Meier survival analysis curves for all-cause mortality. PSM, propensity score match.

Kaplan–Meier analyses showed that the cumulative incidence of 7-day, 30-day, and 360-day all-cause mortality were significantly higher in the no beta blockers group both before and after PSM (all log-rank p < 0.001, Figure 4).

### 3.3 Cox regression analysis

Before PSM, univariate Cox regression analysis (Table 2 Model 1) revealed that beta blocker administration within 24 h of ICU admission showed significant correlation with reduced 7-day (HR = 0.46 95%CI: 0.40, 0.53, p < 0.001), 30-day (HR = 0.58 95%CI: 0.53, 0.64, p < 0.001), and 360-day (HR = 0.69 95%CI: 0.65, 0.73, p < 0.001). The models were further adjusted for age and sex (Model 2) as well as multivariate adjustment (Model 3); it showed that patients in an early use of beta blockers group had a lower risk of 7-day (HR = 0.52 95%CI: 0.44, 0.61, p < 0.001), 30-day (HR = 0.68 95%CI: 0.61, 0.75, p < 0.001), and 360-day (HR = 0.78 95%CI: 0.73, 0.83, p < 0.001) all-cause mortality (Table 2). After PSM, Cox regression analysis showed early administration of beta blockers was associated with significantly reduced 7-day (HR = 0.52 95%CI: 0.44, 0.62, p < 0.001), 30-day (HR = 0.70 95%CI: 0.63, 0.78, p < 0.001), and 360-day (HR = 0.83 95%CI: 0.77, 0.89, p < 0.001) all-cause mortality (Table 2).

**TABLE 2:** Association of beta blocker administration within 24 h with 7-day and 30-day all-cause mortality.

	7-day all-cause mortality			30-day all-cause mortality			360-day all-cause mortality		
	HR	95% CI	p-value	HR	95% CI	p-value	HR	95% CI	p-value
Before PS match									
Model 1	0.46	0.40, 0.53	<0.001	0.58	0.53, 0.64	<0.001	0.69	0.65, 0.73	<0.001
Model 2	0.44	0.38, 0.51	<0.001	0.56	0.51, 0.62	<0.001	0.66	0.62, 0.71	<0.001
Model 3	0.52	0.44, 0.61	<0.001	0.68	0.61, 0.75	<0.001	0.78	0.73, 0.83	<0.001
After PS match	0.52	0.44, 0.62	<0.001	0.70	0.63, 0.78	<0.001	0.83	0.77, 0.89	<0.001

Model 1: unadjusted; Model 2: adjusted by age and gender; Model 3: adjusted by age, gender, race, weight, systolic blood pressure, heart rate, respiratory rate, hematocrit, hemoglobin, platelet, white blood cell, red blood cell distribution, blood urea nitrogen, creatinine, potassium, cerebrovascular disease, chronic pulmonary disease, liver disease, diabetes, chronic kidney disease, cancer, acute myocardial infarction, atrial fibrillation, sepsis, and sofa score.

### 3.4 Subgroup analysis

**FIGURE 5:**
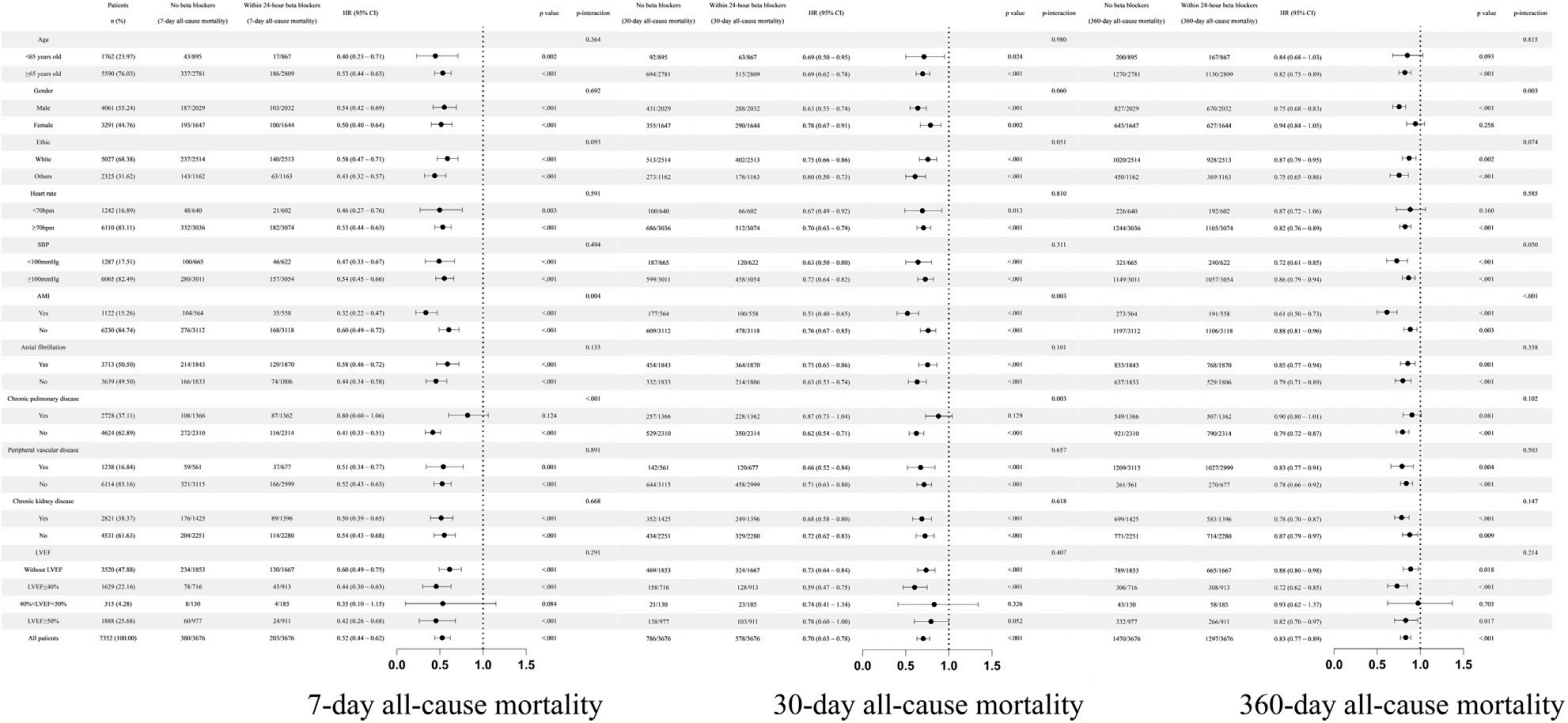
Subgroup analysis. SBP, systolic blood pressure; LVEF, left ventricular ejection fraction.

Subgroup analysis was conducted in different subgroup patients (Figure 5). For 7-day all-cause mortality, the effect of within 24-h use of beta blockers was found consistent in most of the different subgroup patients except that in with or without acute myocardial infarction (p-interaction = 0.004, Figure 5), but the effect was only numerically different (for patients without acute myocardial infarction: HR = 0.60 95%CI: 0.49, 0.72, p < 0.001; for patients with acute myocardial infarction: HR = 0.32 95%CI: 0.22, 0.47, p < 0.001). However, the effect of 24-h beta blockers was different in with or without chronic pulmonary disease subgroup patients (p-interaction<0.001, for patients without chronic pulmonary disease: HR = 0.41 95%CI: 0.33, 0.51, p < 0.001; for patients with chronic pulmonary disease: HR = 0.80 95%CI: 0.60, 1.06, p = 0.124). Similar results were found in 30-day all-cause mortality; however, for 360-day all-cause mortality, the interaction was found in sex subgroup (p-interaction = 0.003, for male patients: HR = 0.75 95%CI: 0.68, 0.83, p < 0.001; for female patients: HR = 0.94 95%CI: 0.84, 1.05, p = 0.258). Moreover, in different LVEF ranges, the effect of beta blockers was consistent without interaction for 7-day, 30-day, and 360-day all-cause mortality (all p-interaction >0.05).

**FIGURE 6:**
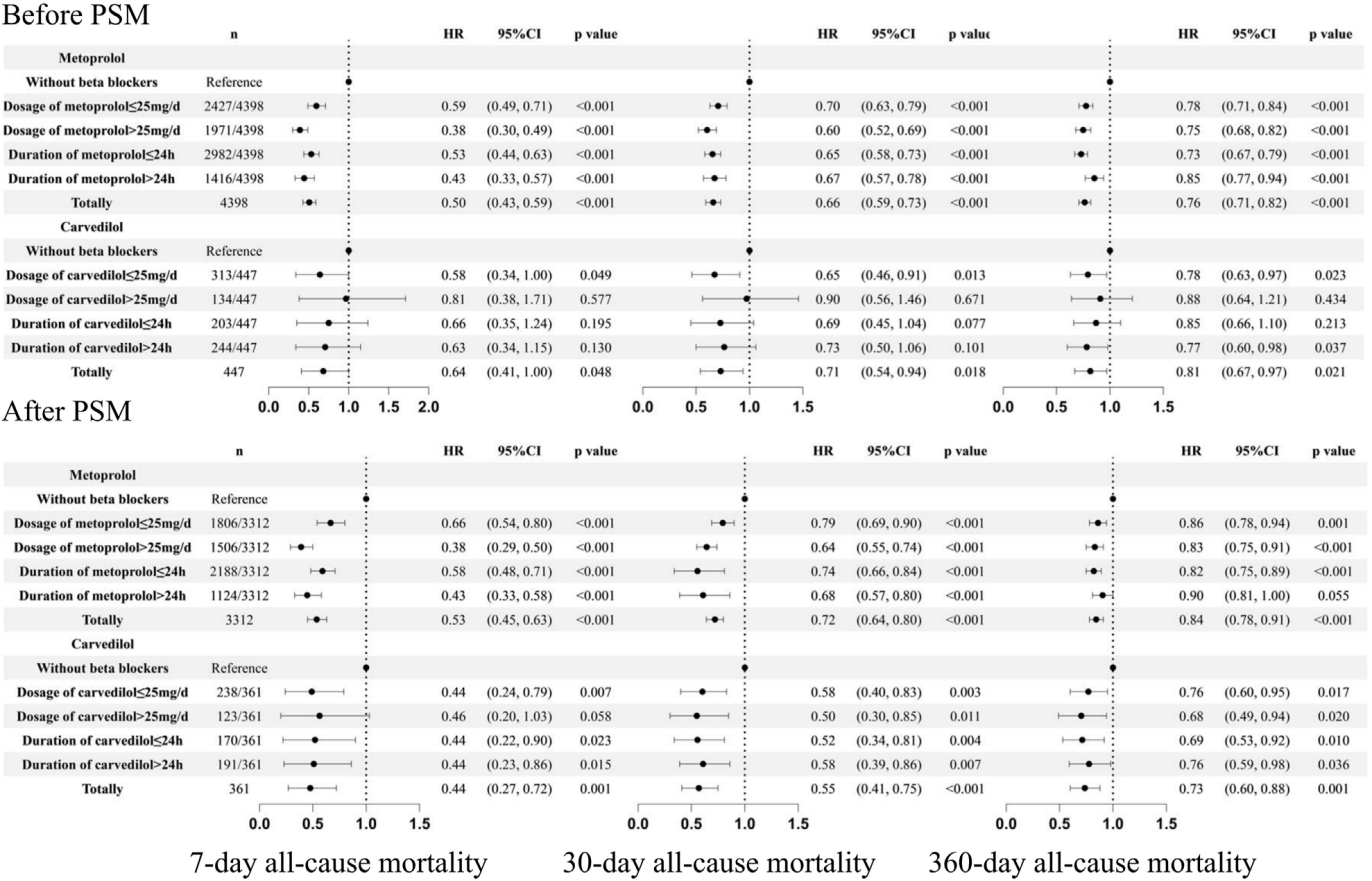
Subgroup analysis based on the dosage and duration of beta blockers. PSM: propensity score match.

Subgroup analysis was also performed in patients who received different types, dosages, and duration of beta-blockers (Asonly four patients received bisoprolol, they were not taken into analysis) (Figure 6). Compared to patients without the use of beta-blockers, different dosages and durations of metoprolol consistently improved patients’ outcomes. For carvedilol, before PSM, dosage >25 mg/day could not improve the prognosis of 7-day (HR = 0.81 95%CI: 0.38, 1.71, p = 0.577), 30-day (HR = 0.90 95%CI: 0.56, 1.46, p = 0.671), and 360-day all-cause mortality (HR = 0.88 95%CI: 0.64, 1.21, p = 0.434). However, after PSM, carvedilol at doses >25 mg/day significantly reduced 30-day (HR = 0.50 95%CI: 0.30, 0.85, p = 0.011) and 360-day all-cause mortality (HR = 0.68 95%CI: 0.49, 0.94, p = 0.020) and showed a trend toward reducing 7-day all-cause mortality (HR = 0.46 95%CI: 0.20, 1.03, p = 0.058), though without statistical significance. Carvedilol at doses ≤25 mg/day, duration ≤24 h or >24 h were consistently associated with reduced risk of 7-day, 30-day, and 360-day all-cause mortality after PSM.

## 4 Discussion

To our knowledge, this is the first study so far to explore whether early administration of beta blockers within 24 h after admission to ICU could provide a survival advantage in critical HF patients. The current study also shows association of early administration of beta blockers with significantly reduced 7-day, 30-day, and 360-day all-cause mortality. It also provided conclusive evidence for the early (within 24 h) use of beta blockers in critical HF patients and is expected to guided prospective studies and clinical practice.

The benefits of beta blockers in patients with chronic HF have been confirmed by multiple randomized controlled trials (Packer et al., 1996; Authors Anonymous, 1999a; Authors Anonymous, 1999b) and are recommended by current guidelines on treating patients with chronic HFrEF (McDonagh et al., 2023; Heidenreich et al., 2022). For acute decompensated HF, several studies demonstrated that early beta blockers initiation could improve the in-hospital outcomes. Abi et al. (Abi et al., 2017) conducted a HF registry in the Middle East, which enrolled 8066 patients admitted with acute decompensated HF, and found that use of beta blockers at admission, compared with those without use, was significantly associated with reduced in-hospital mortality (3.6% vs 14.4%, HR = 0.23, 95% CI, 0.18, 0.61, p = 0.001). However, this cohort study started in 1991 when beta-blocker therapy was not widely implemented in HF patients. Moreover, one-third of the patients were accompanied by acute coronary syndrome (ACS), and the benefit of beta blockers might be through reducing acute ischemia in ACS patients. Therefore, the findings of this study are not universally applicable. Another study from the Italian Survey on Acute Heart Failure also confirmed the beneficial effect of beta blockers in worsening HF patients, the in-hospital mortality of patients receiving beta-blockers at admission and continuing during hospitalization was significantly lower than that in patients not receiving beta-blockers (2.8% vs 10.1%, HR = 3.28, 95% CI 1.47, 7.32, p = 0.004) (Orso et al., 2009). This study also has obvious bias. It is an old study since 2003 and the patients in this study were relatively young, which limited its clinical application. Additionally, a more recent study conducted by Tamaki et al. (Tamaki et al., 2021) demonstrated that acute decompensated HF patients treated with beta-blockers at admission had significantly lower in-hospital mortality rates (4.4% vs 7.6%, p < 0.001) and the adjusted odds ratio of patients with versus without beta blockers at admission was 0.41 (95% CI, 0.27, 0.60, p < 0.001) for in-hospital death. A recent meta-analysis involved eight cohort studies with 16,639 acute HF or cardiogenic shock patients confirmed that early beta blockers initiation provided a survival advantage, including the in-hospital composite endpoints (RR = 0.42; 95% CI, 0.30, 0.58, p < 0.001), in-hospital all-cause mortality (RR = 0.43; 95% CI, 0.31, 0.61, p < 0.001), discharge mortality (RR = 0.51, 95% CI, 0.41, 0.63, p < 0.001), and rehospitalization (RR = 0.57; 95% CI, 0.44, 0.74, p < 0.001) (Sinardja et al., 2024). Meanwhile, this meta-analysis also demonstrated that early beta blocker initiation in acute HF patients was safe (Sinardja et al., 2024). However, all of the aforementioned studies could not focus on critical HF patients, who had significantly impaired cardiac function, severely reduced cardiac contractility, and severe systemic hypoperfusion (Crespo-Leiro et al., 2018). Whether critical HF patients could benefit from early beta blocker use remains poorly understood, and our study corroborated and extended previous findings, indicating that beta blocker administration within 24 h after admission to ICU could provide a survival advantage in critical HF patients.

Though still unclear, the mechanism behind the early use of beta blockers and better prognosis can be explained through the following aspects. First, sympathetic overactivity is often prevalent in HF, activated sympathetic nervous system increases both preload and afterload by constricting the arterial and venous (Bruning et al., 2021; Mentz and O'Connor, 2016), while beta-blockers could attenuate the adverse effect of sympathetic overactivity. Furthermore, sympathetic overactivity causes increased heart rate, which further leads to diastolic shortening and insufficient myocardial blood supply, beta blockers reduce heart rate and prolong diastole by blocking beta-receptors, thereby improving myocardial blood supply and prognosis (Khan et al., 2023; Kezerashvili et al., 2012). Earlier studies have demonstrated that in both HFrEF patients and HF animal models, beta blockers can effectively inhibit neurohumoral activation, improve ventricular remodeling, and increase ejection fraction (Cleland et al., 2018; Sun et al., 2005). Second, beta-blockers may play an important role in antioxidant and anti-inflammatory as beta blockers can downregulate inflammatory pathways, reduce the production of reactive oxygen species, and maintain neurohormonal stability (Rossi et al., 2022; Nakamura et al., 2011). The antioxidant and anti-inflammatory properties of beta blockers decrease the strain on the heart, lower myocardial oxygen demand, and prevent the development of malignant arrhythmia (Shah et al., 2019). In addition, beta-blockers can improve ventricular function, reduce chamber dilation, and improve overall cardiac performance (Cleland et al., 2018; Enzan et al., 2021). Therefore, early beta blocker initiation may provide a survival advantage in critical HF patients.

The present study has important clinical implications. It is a pioneer study to explore whether the early use of beta blockers can improve outcomes in critically ill HF patients and confirms that beta blocker administration within 24 h of ICU admission is associated with a reduced risk of mortality in this population. In clinical practice, critically ill HF patients often experience more severe cardiac dysfunction and circulatory instability (Crespo-Leiro et al., 2018). Due to the negative inotropic effects of beta-blockers (Tamaki et al., 2021), clinicians may show reluctance to prescribe these beta blockers, which results in delays or avoidance of their use. However, the present study findings may help alleviate these concerns by demonstrating the potential benefits of early beta-blocker administration. Furthermore, subgroup analysis showed that the beneficial effects of early beta-blocker use were consistent across different subgroups, including patients with relatively lower systolic blood pressure (<100 mmHg) and heart rate (<70 bpm), two of the most common reasons for withholding beta-blockers (McDonagh et al., 2023; Heidenreich et al., 2022). However, caution is warranted in critically ill HF patients with concomitant chronic pulmonary disease, because the current study indicated that these patients did not benefit from beta blockers. This may be due to the potential adverse effects of beta blockers on airway function (Jabbour et al., 2010). In addition, subgroup analysis revealed an interaction between gender and the use of beta blockers in 360-day all-cause mortality, with no significant benefit observed in women. However, no interaction was observed in 7-day or 30-day all-cause mortality. The gender difference observed at 360 days may be due to sample size effects, and further research is needed to confirm whether such a difference truly exists.

More importantly, the present study also conducted subgroup analyses based on LVEF and demonstrated that the benefits of beta blockers were consistent across different LVEF groups. Our study also shows that the effects of different beta blockers were similar, which corroborates previous evidence for beta blockers in patients with HFrEF (McDonagh et al., 2023; Heidenreich et al., 2022). Moreover, previous studies have shown association of cardiac remodeling in HF patients with the dosage and the duration of use of beta-blockers (Marti et al., 2019; Bristow, 2000). The current study also suggested that in patients treated with metoprolol, higher dosage, and longer treatment durations were associated with a more significant reduction in the risk of death. However, because of the substantial individual variability in the tolerance of beta blockers in critically ill HF patients, treatment should be administered individually. These findings provided valuable evidence strengthening the early use of beta blockers in critically ill HF patients and offered key insights to help clinicians identify critical HF patients who may most likely benefit from this early administration of beta blockers. Additionally, our study also offered preliminary evidence for the design of prospective randomized controlled trials to assess whether early use of beta blockers benefits critically ill HF patients.

Importantly, this study has some limitations . First, there is considerable individual variation in the dosage and treatment duration of beta-blocker use. We divided patients into two groups based solely on data from the majority of patients, which may, to some extent, affect statistical efficiency and the accuracy of the results. Moreover, among the beta-blockers, approximately 90% of patients were treated with metoprolol, while fewer patients used carvedilol or bisoprolol, which may impact the statistical results. Furthermore, the MIMIC-IV database could not provide specific indications for beta-blocker use in individual patients. Second, LVEF is an important indicator for assessing cardiac function and is associated with prognosis. However, not all patients in the MIMIC database had available LVEF values. Nevertheless, among the patients with available LVEF data, we demonstrated that the benefits of beta-blocker use were consistent across different ranges of LVEF, and there was no interaction between patients with and without LVEF values. Furthermore, after grouping by LVEF, the number of HFmEF patients was relatively small, which may also affect statistical power. Third, The MIMIC database only includes all-cause mortality as an endpoint and lacks endpoints such as cardiovascular mortality, readmission due to heart failure, major cardiovascular adverse events, length of hospitalization, hospitalization expenses, and the safety of beta blockers. Fourth, the severity of symptoms may influence clinicians; decisions to administer beta-blockers within 24 h; also, sufficient data could not be collected on the severity of patient symptoms, which may impact the results of our study. In addition, as a retrospective study, potential biases and factors were not well-controlled and could impact the outcomes, although PSM analysis was used to reduce the selection bias. Therefore, to rationally interpret the findings, and conduct more prospective studies, especially randomized controlled studies with larger samples are needed to confirm our findings.

## 5 Conclusion

Beta-blockers administration within 24 h after admission to ICU provided a survival advantage in critical HF patients and was significantly associated with reduced 7-day, 30-day, and 360-day all-cause mortality. Randomized controlled studies are warranted to confirm this finding, the future research may focus on the dose-effect evaluation of beta-blockers, and the other outcomes including cardiovascular mortality, readmission due to HF, major cardiovascular adverse events, and the safety of beta blockers.

## Data Availability

Publicly available datasets were analyzed in this study. These data can be found here: the American Medical Information Mart for Intensive Care (MIMIC)-IV (version 3.0) database.
